# Function of Centriolar Satellites and Regulation by Post-Translational Modifications

**DOI:** 10.3389/fcell.2021.780502

**Published:** 2021-11-23

**Authors:** Clotilde C. N. Renaud, Nicolas Bidère

**Affiliations:** CNRS, CRCINA, INSERM, Université de Nantes, Nantes, France

**Keywords:** centriolar satellites, ubiquitin, phosphorylation, post-translational modification (PMT), centrosome, primary cilia

## Abstract

Centriolar satellites are small membrane-less granules that gravitate around the centrosome. Recent advances in defining the satellite proteome and interactome have unveiled hundreds of new satellite components thus illustrating the complex nature of these particles. Although initially linked to the homeostasis of centrosome and the formation of primary cilia, these composite and highly dynamic structures appear to participate in additional cellular processes, such as proteostasis, autophagy, and cellular stress. In this review, we first outline the main features and many roles of centriolar satellites. We then discuss how post-translational modifications, such as phosphorylation and ubiquitination, shape their composition and functions. This is of particular interest as interfering with these processes may provide ways to manipulate these structures.

## Introduction

**FIGURE 1:**
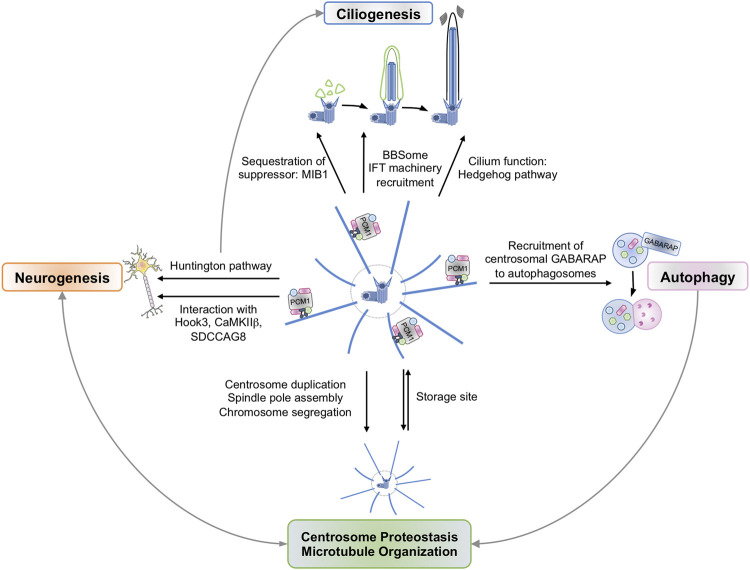
Centriolar Satellites participate in a variety of cellular Functions. Centriolar satellites shuttle back and forth on microtubules to control the abundance of centrosomal proteins. This could explain the role of satellites in centrosome duplication and separation, spindle pole assembly, and chromosome segregation. These site storage properties have also been linked to neurogenesis. To preserve centrosome organization and stability, centriolar satellites can be degraded in GABARAP-dependent autophagosomes and *via* the ubiquitin-proteasome system (UPS). Conversely, the satellite component PCM1 regulates GABARAP level and GABARAP-dependent autophagic flux. Centriolar satellites also participate in primary cilium formation and function. Finally, satellites shape cellular proteostasis and thereby may participate in functions beyond centrosomal and ciliary functions.

The centrosome is an organelle that consists of two structural sub-components: A pair of orthogonal centrioles, surrounded by a cloud of pericentriolar material. This serves as the primary microtubule organizing center (MTOC) in a large variety of animal cells and acts as a multifunctional platform for numerous signaling processes such as cell cycle progression, mitosis, and ciliogenesis. With the democratization of electron microscopy in the second half of the 20th century, several groups observed electron-dense particles of 70–100 nm in diameter surrounding the centrosome, which they coined centriolar satellites (Bessis and Breton-Gorius, 1958; Bernhard and de Harven, 1960; de-Thé, 1964). However, these small non-membranous structures remained enigmatic until the mid-1990s with the discovery of the Pericentriolar Material 1 protein (PCM1) in human cells using human autoimmune serum (Balczon et al., 1994). By cloning the *Pcm1* ortholog from *Xenopus* and demonstrating its presence within these electron-dense granules by immunogold electron microscopy, Kubo and others subsequently established PCM1 as a *bona fide* centriolar satellite marker in cells (Kubo et al., 1999). It was further discovered that most centriolar satellite components display diffuse motility along microtubules in a dynein-dependent manner, are required for the assembly of centrosomal proteins, and undergo cell cycle-dependent assembly and disassembly (Kubo et al., 1999; Dammermann and Merdes, 2002; Conkar et al., 2019). Centriolar satellites were also shown to play a role in the cargo trafficking to the centrosome and the primary cilium, thereby orchestrating ciliogenesis (Figure 1) (Tollenaere et al., 2015a).

The founding centriolar satellite member PCM1 is a large (>230 kDa) scaffold protein, crucial for the maintenance and function of this multiprotein network (Wang et al., 2016). Accordingly, mutation or depletion of PCM1 disassembles the centriolar satellites and leaves cells devoid of satellites. PCM1 is composed of 8 coil-coiled domains that allow its self-aggregation and the binding of other satellite components, including CEP131, CEP290, OFD1, or MIB1 (Wang et al., 2016). The initial view was that, for a protein to be a part of centriolar satellites, three criteria must be met: 1) it localizes in the vicinity of the centrosome, 2) it physically interacts with PCM1, and 3) it is redistributed to the cytoplasm upon microtubule network disruption (Kubo et al., 1999; Dammermann and Merdes, 2002; Kubo and Tsukita, 2003). This classification led to the identification of >65 centriolar satellite resident proteins. However, the situation is likely to be far more complicated. Strikingly, components of satellites are not necessarily encircling the centrosome and can be also found around the nucleus, at basal bodies, or dispersed in the cytoplasm, depending on the cell type and differentiation state (Kubo and Tsukita, 2003; Vladar and Stearns, 2007; Srsen et al., 2009; Odabasi et al., 2020). Two recent studies investigating the proteome and interactome of centriolar satellites underscored the composite and highly complex nature of these structures (Gheiratmand et al., 2019; Quarantotti et al., 2019). This work led to the identification of 223 PCM1-associated proteins and the characterization of 660 proteins that interact with 22 satellite proteins, with a 40% overlap (Gheiratmand et al., 2019; Quarantotti et al., 2019). Hence, different subgroups of centriolar satellite proteins may exert distinct functions within cells (Odabasi et al., 2020). Despite a strong effort put into elucidating the composition and interactions between satellite-associated proteins [for recent exhaustive reviews, please see (Tollenaere et al., 2015a; Hori and Toda, 2017; Odabasi et al., 2020; Prosser and Pelletier, 2020)], the molecular basis for centriolar satellites assembly and localization remains enigmatic. Here, we describe the main roles ascribed to centriolar satellites and discuss how post-translational modifications regulate their organization and functions.

## Functions of Centriolar Satellites

### Participation in the Homeostasis of Centrosome

Centriolar satellites gravitate around the centrosome and are intimately linked to this organelle. Comprehensive analyses of the centriolar satellite proteome revealed that a significant subset of proteins is shared between centrosome and centriolar satellites. For instance, almost half of the known centrosome proteome was detected in centriolar satellites in chicken cells (Quarantotti et al., 2019). In human cells, an overlap of approximately 40% was found between the interactome of the centriolar satellites and that of the centrosome (Gheiratmand et al., 2019). Centriolar satellites have been suggested to function as temporary storage sites to finely tune the abundance of centrosomal proteins (Figure 1). In line with this, centriolar satellites allow the transport of selected proteins, including Nek2A and CaMKII*β*, to the centrosome whilst limiting others (Hames et al., 2005; Puram et al., 2011). This PCM1-dependent modulation of CaMKII*β* abundance at the centrosome was proposed to control its function in dendrite patterning and neuronal connectivity in mammalian brains (Puram et al., 2011). In the brain, PCM1 also interacts with the centrosome-associated protein Hook3 (Ge et al., 2010). Such binding allows proper protein assembly at the centrosome and thereby prevents overproduction of neurons and premature depletion of the neural progenitor pool in the developing cortex (Ge et al., 2010). Further supporting the idea that centriolar satellites “feed” the centrosome, several centriolar satellite components were linked with proper centrosome duplication, separation, spindle pole assembly, and chromosome segregation (Figure 1) (Oshimori et al., 2009; Kim and Rhee, 2011). This interplay between satellites and centrosome relies on the capacity of centriolar satellites to crawl along microtubules anchored to the centrosome. The physical coupling between centriolar satellites and microtubule-associated motor proteins such as dynein are mediated by several satellite components, including BBS4 (Kim et al., 2004), Par6*α* (Kodani et al., 2010), or CEP290 (Kim et al., 2008). In keeping with this, several studies support a role for centriolar satellites in microtubule organization, further drawing a parallel with the MTOC function of the centrosome. First, the abundance of centrin, pericentrin, and ninein, known regulators of microtubule anchorage to centrosomes, is reduced in the absence of PCM1, thereby disturbing the microtubule network (Dammermann and Merdes, 2002). Similarly, the silencing of satellite residents BBS4 or CEP290 also impairs microtubules organization (Dammermann and Merdes, 2002; Kim et al., 2004; Kim et al., 2008). Conversely, the microtubule network is essential to maintain centriolar satellites integrity, and microtubules depolymerization drives the dispersion of centriolar satellites from the pericentriolar region to the entire cytoplasm (Dammermann and Merdes, 2002).

### Orchestration of Ciliogenesis

The primary cilium is a small microtubule-based structure that protrudes from the cell body and acts as a “sensing antenna” to detect and integrate various extracellular signals, such as proteins, low molecular weight chemicals, light, and mechanical stimuli (Malicki and Johnson, 2017). The primary cilium is composed of a microtubule-based scaffold core structure called axoneme, surrounded by a ciliary membrane that is continuous with the plasma membrane. The axoneme originates from the basal body, which is a differentiated centrosome. The formation of primary cilium occurs during the early G1 or G0 phases, whereas its disassembling precedes cell division. Defects in primary cilia formation can lead to a wide range of inherited developmental and degenerative diseases, called ciliopathies. Because of the pleiotropic roles of primary cilia, ciliopathies are associated with a broad spectrum of symptoms, from polydactyly, retinal degeneration, and obesity to cystic kidney disease, and neurodevelopmental defects (Waters and Beales, 2011; Malicki and Johnson, 2017). There is now a growing body of literature that supports a role for centriolar satellites in ciliogenesis. First, human retina pigment epithelial (RPE-1) cells knockout for PCM1 by CRISPR/Cas9 or silenced for various centriolar satellite proteins display impaired ciliogenesis (Kim et al., 2008; Lee and Stearns, 2013; Klinger et al., 2014; Staples et al., 2014; Wang et al., 2016; Conkar et al., 2017; Odabasi et al., 2019). The deletion of PCM1 in the kidney epithelial cell line IMCD3 also drastically reduces primary cilia assembly and function (Odabasi et al., 2019). Moreover, mutations in genes encoding for centriolar satellite components were reported to cause failure or dysfunction of primary cilia in ciliopathies (Valente et al., 2006; Hori and Toda, 2017; Prosser and Pelletier, 2020). Of note, acute or chronic deletion of centriolar satellites may give different phenotypes likely due to a compensation phenomenon. This is the case of CEP131, whose targeting by siRNA leads to defect in ciliogenesis, whereas gene deletion has no effect (Hall et al., 2013).

How centriolar satellites participate in ciliogenesis is complex, as they likely contribute to both primary cilium formation and function (Figure 1). For instance, centriolar satellites were linked to the recruitment of the BBSome, a ciliary trafficking multi-protein complex, to the primary cilium. Five members of the BBSome (BBS2, BBS4, BBS7, BBS8, and BBS9) are part of the satellite proteome (Quarantotti et al., 2019). BBS4 was further shown to interact with PCM1, CEP131, and CEP290, and its recruitment to the basal body is tightly controlled by CEP131 (Chamling et al., 2014). In addition, the centriolar satellite component SSX2IP is a factor for microtubule anchoring to both centrosome and basal body (Hori et al., 2014). Accordingly, SSX2IP is required for efficient recruitment of the BBSome to the cilium, and its silencing causes shortening of cilium length (Klinger et al., 2014). Centriolar satellites also sequester negative regulators of ciliogenesis away from the basal body during cilia formation, such as the E3 Ubiquitin ligase Mindbomb 1 (MIB1) (Wang et al., 2016). Moreover, centriolar satellites regulate the recruitment of the intraflagellar transport (IFT) machinery, which is essential for the movement of complexes along the axoneme and for the sorting of signaling receptors to the ciliary membrane (Malicki and Johnson, 2017). For example, OFD1 is necessary for distal appendage formation, IFT88 recruitment, and primary cilia formation, and its removal from centriolar satellites by autophagy promotes ciliogenesis (Tang et al., 2013). In keeping with this, OFD1 mutations in humans cause a dysfunction in the formation of primary cilia and disorders such as oral-facial-digital syndrome, Joubert syndrome, and nephronophthisis-related ciliopathies. Finally, centriolar satellites have also been linked to the Hedgehog pathway, a signal transduction pathway that requires functional primary cilia (Prosser and Pelletier, 2020). In cells lacking satellites but still forming cilia, the accumulation of Hedgehog receptor Smoothened is reduced in cilia and this prevents the activation of target gene expression (Goetz et al., 2009; Wheway et al., 2018; Odabasi et al., 2019; Akhshi and Trimble, 2021). Altogether, these examples illustrate the crucial role played by centriolar satellites during ciliogenesis.

### Regulation of Cellular Proteostasis

With our improved understanding of the role of centriolar satellites in ciliogenesis, new paradigms linking these still enigmatic structures to functions beyond the formation of primary cilia have emerged. Several reports now connect satellites to the maintenance of proteostasis (Figure 1). For instance, the loss of PCM1 causes a decrease in the abundance of several centriolar satellite components (Villumsen et al., 2013; Wang et al., 2016). By contrast, MIB1 protein levels are increased in satellite-depleted cells (Villumsen et al., 2013; Wang et al., 2016; Douanne et al., 2019). Recently, Odabasi *et al* carried out a quantitative transcriptomic and proteomic profiling in satellite-depleted epithelial IMCD3 cells (Odabasi et al., 2019). Although no overt change was observed in the global transcriptome, the proteome was significantly altered, as nearly 300 proteins were downregulated while 300 were upregulated. In addition to categories related to “centrosome”, “cell proliferation” and “microtubules”, cellular processes related to the actin cytoskeleton, cell migration and adhesion, endocytosis, neuronal processes, and metabolic processes were perturbed, suggesting functions beyond the centrosome/cilium complex (Odabasi et al., 2019). Further echoing a role for centriolar satellites in the control of proteostasis, the loss of BBS4 or OFD1 alters the abundance of transducers of the Wnt, Hedgehog, or Notch signaling pathways (Gerdes et al., 2007; Liu et al., 2014). From a molecular standpoint, BBS4 and OFD1 physically interact with centrosomal subunits of the proteasome and control its composition and activity (Liu et al., 2014). This function ascribed to centriolar satellites is however not restricted to the centrosomal and ciliary contexts, as BBS1, BBS4, or OFD1 also govern the proteasomal degradation of the NF-κB inhibitor IκB*α*, and thereby the activity of the transcription factor (Liu et al., 2014). Collectively, these examples highlight the involvement of centriolar satellites in the control of proteostasis and the need to further explore their contribution in functions beyond the centrosome and ciliogenesis.

## Post-Translational Modifications Tightly Regulate Centriolar Satellites

The dynamic and disparate nature of centriolar satellites implies *de facto* the existence of tight regulation processes. Over the last decade, a subtle choreography of phosphorylation, ubiquitination, and selective degradation has been shown to shape these structures.

### Regulation by Phosphorylation

**FIGURE 2:**
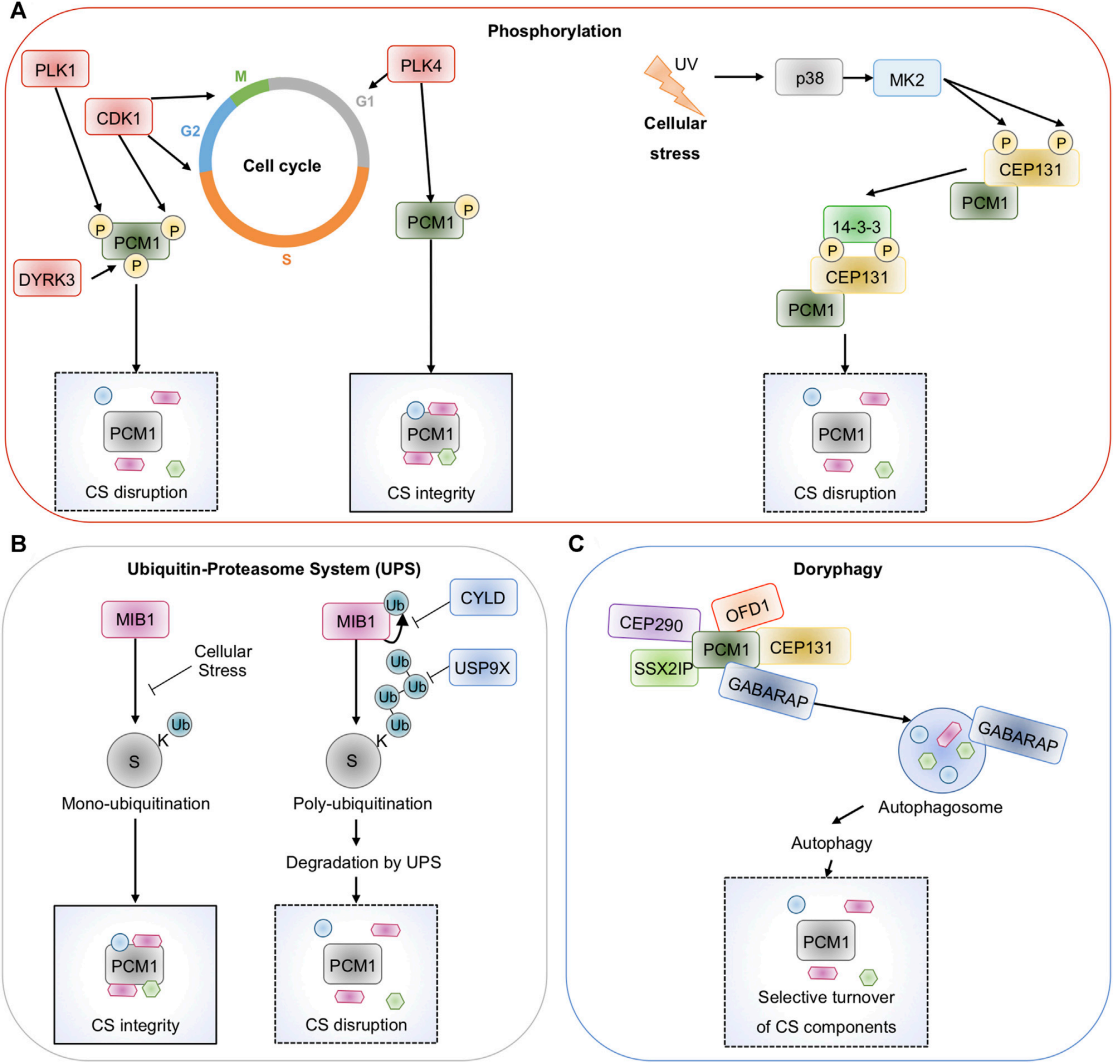
Post-translational control of Centriolar Satellites. **(A)**, Centriolar satellites are drastically reorganized during mitosis. Kinases including PLK1, CKD1, DYRK3, and PLK4 phosphorylate the scaffold PCM1 and control its properties and location. In response to cellular stresses such as UV radiation, heat shock, or transcription block, MK2 phosphorylates CEP131 to allow binding of 14-3-3 proteins and satellites dispersal. **(B)**, The E3 ligase MIB1 plays counterposing role in the homeostasis of centriolar satellites. By stapling mono-ubiquitin onto several components, MIB1 maintains satellites integrity. MIB1 also adds poly-ubiquitin chains to PCM1 and CEP131 and marks them for proteasomal degradation. This is counteracted by the deubiquitinases CYLD and USP9X. **(C)**, PCM1 binds to the autophagy regulator GABARAP and allows the selective turnover of satellites components in a process called doryphagy.

Centriolar satellites are versatile structures that disappear during mitosis and reappear when cells proceed to interphase (Kubo et al., 1999; Dammermann and Merdes, 2002; Kubo and Tsukita, 2003). Yet, these drastic changes occur without affecting the overall abundance of PCM1 and other centriolar satellite elements, thus suggesting that they are regulated through post-translational modifications. The phosphorylation of PCM1 by an array of kinases was proposed to contribute to the cell-cycle-dependent remodeling of centriolar satellites. First, PCM1 phosphorylation on its Serine residue 372 by PLK4 during the G1 phase is essential for its dimerization and scaffolding activity, allowing for centriolar satellites integrity, centriole duplication, and ciliogenesis (Hori et al., 2016). In addition to PCM1, PLK4 phosphorylates CEP131 on S78, thereby maintaining centriolar satellites stability (Denu et al., 2019). PCM1 also undergoes phosphorylation during the G2 phase and mitosis. Notably, Cyclin-dependent kinase 1 (CDK1) phosphorylation of PCM1 on the Threonine in position 703, prior to entry in mitosis, allows the recruitment of PLK1, which then phosphorylates PCM1 on the S110 residue (Wang et al., 2013). Accordingly, PLK1 is involved in primary cilia resorption, further illustrating the relationship between ciliogenesis, centriolar satellites dynamic, and cell cycle regulation (Wang et al., 2013). Moreover, the kinase activity of the dual-specificity tyrosine-phosphorylation-regulated kinase 3 (DYRK3) has recently been demonstrated to drive the dispersal of several membrane-less structures including centriolar satellites during mitosis (Figure 2A) (Rai et al., 2018).

In 2013, Villumsen et al elegantly showed that phosphorylation of some centriolar satellite proteins also occurs in cells exposed to external cues such as UV radiation, heat shock, and transcription blocks (Villumsen et al., 2013). These cellular stresses drive a drastic relocation of a number of satellite components to the cytosol, in a p38*α*-dependent manner. It was subsequently demonstrated that p38-activated downstream kinase MK2 phosphorylates two residues on CEP131 (S47 and S78), thus generating direct binding sites for 14-3-3 proteins, which isolates CEP131 into the cytosol and prevent the formation of new centriolar satellites (Figure 2A) (Tollenaere et al., 2015b).

### Regulation by the Ubiquitin-Proteasome System

Ubiquitination refers to the post-translational modification, by which ubiquitin is added to an acceptor Lysine residue on a target substrate. Ubiquitination can consist of the binding of one ubiquitin moiety (mono-ubiquitination) or of poly-ubiquitin chains, as ubiquitin itself is a substrate of ubiquitination at 8 different positions (Clague et al., 2019). Depending on the cellular context, this vast combination of chains, often referred to as the ubiquitin code, changes the fate of a substrate, *ie* abundance, activation, or location (Clague et al., 2019). Given its crucial role in nearly all cellular processes in Eukaryotes, it is not a surprise that ubiquitination also tightly controls centriolar satellites. A first link came with the discovery that blocking the Ubiquitin-Proteasome System (UPS) results in the accumulation of various centrosome proteins at the pericentriolar material and to an enrichment of PCM1 containing centriolar satellites, consistent with a constant turnover of pericentriolar material proteins (Didier et al., 2008). A cornerstone is MIB1. This E3 ligase is partly found at the centrosome and the centriolar satellites and was reported to mark a specific subset of centriolar satellite proteins with mono-ubiquitin or poly-ubiquitin chains (Figure 2B) (Prosser and Pelletier, 2020). Known satellite substrates of MIB1 include PCM1, CEP131, CEP290, CCDC14, KIAA0753, or OFD1. MIB1-mediated poly-ubiquitination of PCM1 and CEP131 was shown to cause their proteasomal degradation, thereby reducing ciliogenesis (Wang et al., 2016). Accordingly, PCM1 and CEP131 were stabilized and ciliogenesis increased without MIB1. Interestingly, PCM1 appears to sequester MIB1 at the centriolar satellites to maintain it inactive (Wang et al., 2016). In keeping with this, MIB1 also regulates the ciliogenesis regulator TALPID3 (also known as KIAA0586) (Wang et al., 2016), and participates *via* PCM1 in the regulation of GABARAP (GABA Type A Receptor-Associated Protein) turnover (Joachim et al., 2017). Finally, MIB1 was also shown to maintain centriolar satellites integrity through the mono-ubiquitination of PCM1, CEP131, and CEP290 (Villumsen et al., 2013). Nevertheless, additional E3 ligases, besides MIB1, are likely involved in the homeostasis of centriolar satellites. Indeed, several conserved E3 ligases were identified in the satellite proteome (Quarantotti et al., 2019). Moreover, the E3 ligases WWP2 and TRIM9 interact with PCM1, but their functions remain unknown (Nielsen et al., 2018). Lastly, UBR5 ubiquitinates the centriolar satellite component CSPP1 (centrosome and spindle pole associate protein 1), which regulates centriolar satellite localization and organization (Shearer et al., 2018).

Ubiquitination is a reversible process, and a family of proteases called deubiquitinating enzymes (DUBs) is in charge of selectively cleaving ubiquitin chains (Clague et al., 2019). Recently, two DUBs were proposed to counteract the UPS at the centriolar satellites (Figure 2B). First, the DUB Cylindromatosis (CYLD) removes ubiquitin chains bound to MIB1 to limit its abundance and activity, thus preventing UPS-mediated degradation of centriolar satellite components (Douanne et al., 2019). Ubiquitin Specific Peptidase 9 X-Linked (USP9X) also physically binds to CEP131 and PCM1, and prevents their proteasomal degradation, hence maintaining centriolar satellite integrity (Li et al., 2017a; Han et al., 2019). Interestingly, USP9X directly trims ubiquitin chains attached to PCM1 and CEP131 to limit their elimination. Yet, removing MIB1 counteracts the effect of USP9X silencing. The central role of USP9X is further illustrated by the identification of mutations underlying ciliopathies in human patients (Reijnders et al., 2016).

### Regulation by Autophagy

In 2013, two pioneer studies revealed an unexpected role for autophagy, a fundamental eukaryotic process that orchestrates the safe disposal of damaged proteins and organelles to lysosomes, in either restricting or promoting the formation of the primary cilium, notably by targeting centriolar satellite components (Pampliega et al., 2013; Tang et al., 2013). More recently, it was discovered that a subset of centriolar satellites also contains the autophagy regulator GABARAP, a mammalian ortholog of the yeast Atg8 required for the maturation of autophagosome during autophagy (Joachim et al., 2017). A seminal work by Joachim et al showed that a pool of PCM1 directly binds GABARAP and protects it from MIB1-directed proteasomal degradation (Figure 2C) (Joachim et al., 2017). Holdgaard et al subsequently revealed that centriolar satellite proteins accumulate in autophagy-deficient cells, forming large and abnormal satellites, thus reinforcing the idea that centriolar satellites are substrates for autophagy (Holdgaard et al., 2019). Interestingly, PCM1 interaction with GABARAP appears essential for PCM1 degradation and loss of centriolar satellites stability. This selective turnover of centriolar satellite components by autophagy, a process named doryphagy, may preserve centrosome organization and stability (Holdgaard et al., 2019).

## Concluding Remarks

Over the last decade, a large body of work has expanded our knowledge of the complex and highly dynamic nature of centriolar satellites. Although it has become clear that these structures play a crucial role in maintaining centrosome homeostasis, in orchestrating ciliogenesis, and cellular proteostasis, the molecular basis behind these functions continues to be elucidated. In addition, whether centriolar satellites are a pool of homogenous or heterogeneous structures remains unclear. In that view, the recent characterization of the global composition of centriolar satellites unveiled hundreds of partners and offers as many putative new layers of regulation to investigate (Gheiratmand et al., 2019; Quarantotti et al., 2019). In keeping with this, 19 kinases, 20 E3 ligases, and 4 DUBs were found in the interactome of centriolar satellites (Gheiratmand et al., 2019). However, centriolar satellites may also regulate the function of these enzymes. Accordingly, centriolar satellites were recently shown to limit the localization, abundance, and activity of the kinase Aurora kinase A (AURKA) (Arslanhan et al., 2021). As a consequence, the activation of AURKA driven by satellite depletion causes defects in ciliogenesis. Finally, whether satellites exert functions beyond ciliogenesis and centrosomal homeostasis is still an open question. Hints may come from the discovery that satellites are rapidly redistributed into the cytosol in response to a range of cellular stresses (Villumsen et al., 2013; Tollenaere et al., 2015b). It would be interesting to investigate whether satellites serve as cellular stress sensors and whether the composition and the interactome of satellites are changed in response to external cues. On a different note, centrosomes were recently shown to function as a platform for the assembly and activity of the NLRP3 inflammasome, a critical component of the innate immune system (Li et al., 2017b; Magupalli et al., 2020). Whether centriolar satellites participate in such signaling pathways is unknown. Future work is therefore required to define the landscape of the functions governed by centriolar satellites.
